# Case Report: Bilateral mandibular buccal bifurcation cysts

**DOI:** 10.12688/f1000research.28000.3

**Published:** 2021-02-19

**Authors:** Ashwag Aloyouny, Hamad Albagieh, Soad Mansour, Fahmy Mobarak

**Affiliations:** 1 Basic Dental Science Department, College of Dentistry, Princess Nourah bint Abdulrahman University, Riyadh, Saudi Arabia; 2 Oral Medicine and Diagnostic Science Department, College of Dentistry, King Saud University, Riyadh, Saudi Arabia; 3 Surgery Department, College of Dentistry, Cairo University, Cairo, Egypt

**Keywords:** Buccal Bifurcation Cysts, Bilateral mandibular cysts, Children, Oral Cyst

## Abstract

Buccal bifurcation cyst (BBC) is a rare inflammatory odontogenic cyst, which commonly affects children in the first decade of life. We report a case of a seven-year-old healthy boy with bilateral BBC, which involved unerupted incomplete permanent mandibular first molars. A review of the literature in English language revealed few similar cases. We reviewed 16 manuscripts of bilateral mandibular BBC, reporting a total of 20 cases since 1970. The clinical features of bilateral mandibular BBC summarized here could assist specialists with an accurate diagnosis and provide patients with optimal management.

## Introduction

Buccal bifurcation cyst (BBC) is a rare inflammatory odontogenic cyst. The first case of BBC was reported in 1983 by Stoneman and Worth ^1^. Children between the age of 4 and 14 years are most commonly affected. In addition, BBC occurs more in the mandible and most likely involves the permanent mandibular first molar. The prevalence of BBC is less than 1% of all odontogenic cysts ^2^. Typically, BBC causes many undesirable oral manifestations such as buccal swelling at the affected area, delayed tooth eruption or partially tooth eruption associated with deep periodontal pockets. In some cases, pain and infection associated with pus could be present ^3^. 

Radiographically, BBC is illustrated as a well-defined radiolucent lesion surrounded by sclerotic rim. The lesion either involves the roots of a partially erupted tooth or surrounds an unerupted tooth; usually the permanent mandibular first molar ^4^. Histopathologically, the BBC cyst wall is lined by a non-keratinized stratified squamous epithelium with inflammatory lymphocytes infiltrate. Surgical excisional procedure is performed for complete removal of the cysts with the involved teeth to reduce the risk of recurrence of the epithelial cysts ^5^.

Here, we report a case of a seven-year-old healthy boy with a chief complaint of painful, slowly growing lower jaw swellings on both right and left sides consistent with bilateral BBC, which involved unerupted incomplete permanent mandibular first molars. A review of the literature revealed few similar cases. We report this case to add to an additional case of bilateral mandibular BBC to the literature.

## Case presentation

A seven-year-old healthy boy presented to the Oral and Maxillofacial Surgery Clinic with a chief complaint of painful, slowly growing lower jaw swellings on both right and left sides. The patient had no past medical, surgical, or dental history. His guardian reported no known drug and food allergies. The guardian also denied having family history of genetic related diseases or syndromes. The swellings caused esthetic disfiguring of the patient’s face, which led to social exclusion.

On examination, extraoral, bilateral asymmetric swellings of the lower part of the face with no lymph node involvement were noted. Intraorally, painful bilateral, hard, bony mandibular swellings covered with normal color mucosa, extending from the mesial aspect of the mandibular second primary molars on both sides including the retromolar areas, was observed. On palpation, the affected site revealed expansion of the buccal cortical plates on both sides. Both mandibular permanent first molars were not clinically erupted.

**Figure 1.**
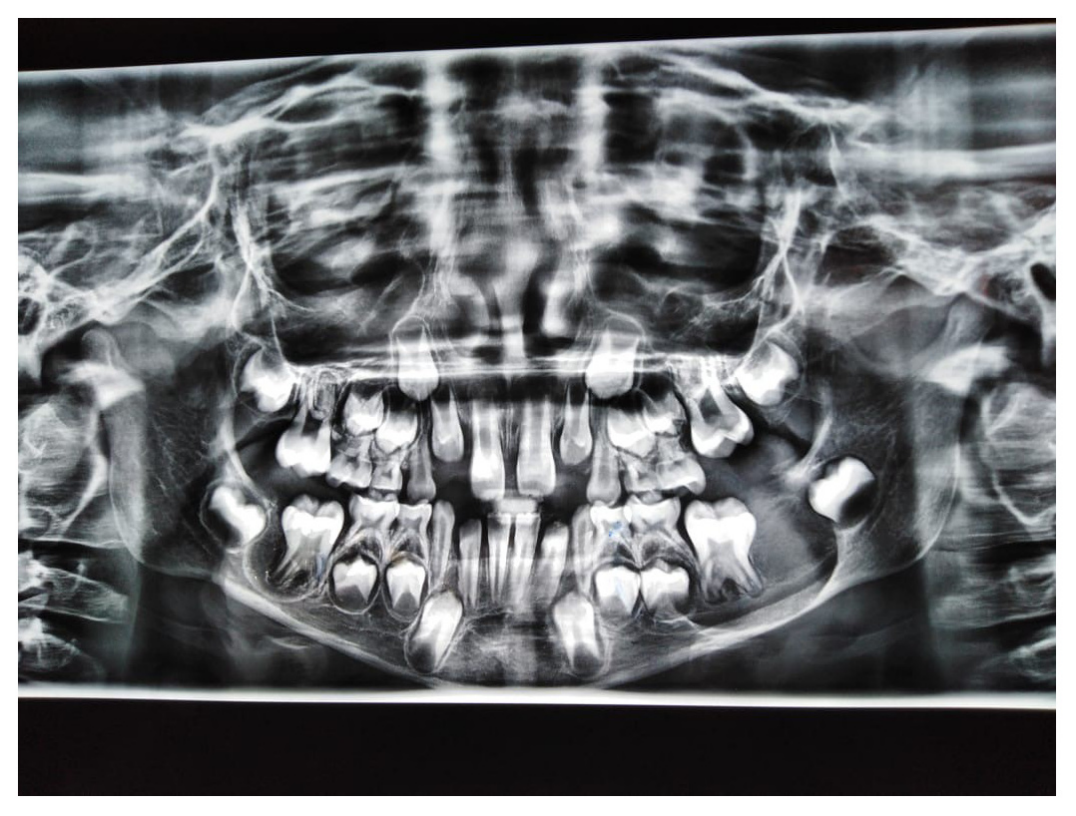
A seven-year-old healthy boy with mixed dentition and bilateral BBC which involved unerupted incomplete permanent mandibular first molars.

A diagnostic panoramic radiograph was taken which illustrated bilateral, well-defined radiolucencies surrounded by sclerotic margins and including the unerupted incomplete permanent mandibular first molars (36 and 46, according to the FDI World Dental Federation Notation). The panoramic image showing right radiolucency measured around 1 cm in its greatest dimension, and involved the unerupted permanent mandibular first molar (46, according to the FDI World Dental Federation Notation), and not involving the inferior mandibular cortical bone and the adjacent areas. On the other hand, the left radiolucency measured around 3cm in its greatest dimension, and involved the unerupted permanent mandibular first molar (36, according to the FDI World Dental Federation Notation), the inferior mandibular cortical bone and the adjacent areas. Moreover, the left radiolucent cyst distally displaced the permanent mandibular second molar tooth bud (37, according to the FDI World Dental Federation Notation) ( Figure 1). Provisional diagnosis of the bilateral lesions suggested bilateral BBC, dentigerous cyst, and paradental cyst.

**Figure 2.**
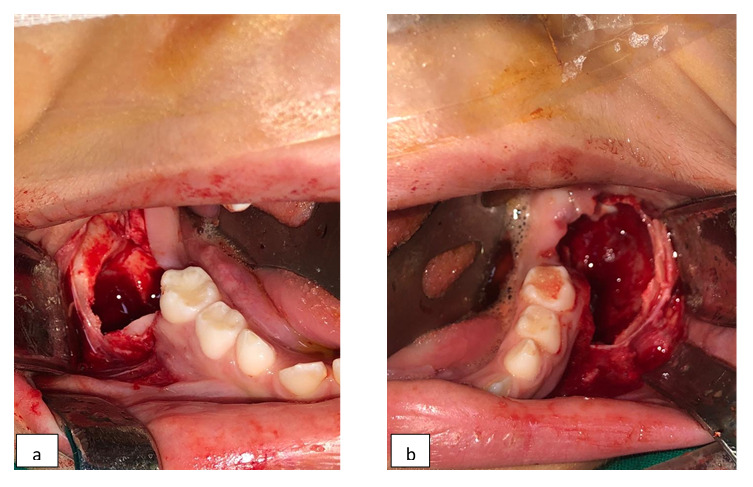
( **a** and **b**) Clinical intraoral photographs show bilateral surgical removal of the bilateral BBC with the involved permanent teeth.

The guardian was informed about the patient’s condition, possible management and complications. Then, a consent form was signed before starting the complete surgical removal of the lesions under general anesthesia. An incision was made, then a buccal mucoperiosteal flap was raised in the mandible at the mandibular permanent first molar area on both right and left sides to expose the bone. The buccal plate was expanded and thinned; especially in the left side. An access to the cyst area was made by performing an ostectomy on both sides. The whole cyst lining was removed with the attached mandibular first molars. Then, the cavity was enucleated and curetted thoroughly ( Figure 2), to reduce the risk of recurrence. The patient was prescribed Ibuprofen 100 mg/5 mL oral suspension four times/day for 3 days in order to reduce the post-surgical pain.

**Figure 3.**
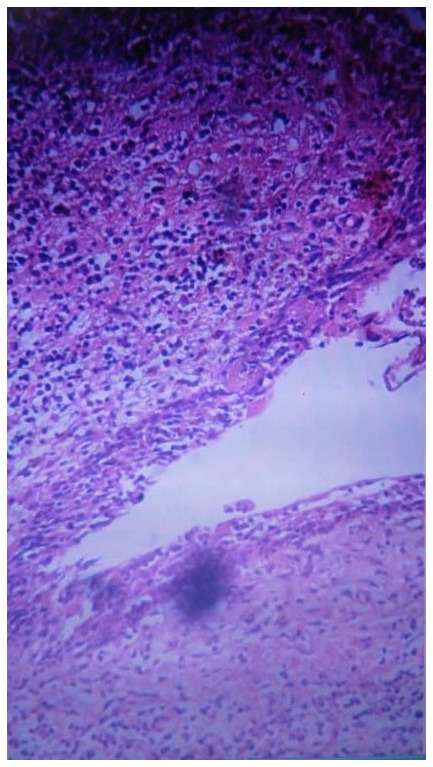
High-power histology shows the cyst wall with chronic inflammatory lymphocytes and two to four cell layers thick of proliferating lining of nonkeratinized stratified squamous epithelium.

The specimens were sent to the histopathology laboratory in two containers. The histopathologic analysis of the multiple serial sections of the two specimens revealed multiple pieces of cyst wall lined by small strip of non-keratinized stratified squamous epithelium with mixed inflammatory reaction and surrounding edematous granulation tissue formed of numerous proliferating capillary type vascular spaces. Also, the specimens under the microscope showed bland looking spindle-shaped fibroblasts and degenerated bony spicules, which was consistent with cystic lesions ( Figure 3). Based on the clinical examination, radiographic interpretation, and histopathologic analysis, the final diagnosis was bilateral BBC.

Six months later, the clinical and radiographic examination of both surgery sites showed signs of gradual healing and resolution and increased bone density in the areas. One year later, no clinical evidence of lesion recurrence, and radiographic imaging illustrated that the affected areas were completely filled up with bone.

## Discussion

Bilateral BBC is an uncommon inflammatory odontogenic cyst. It usually arises from the buccal side of the mandibular permanent first molar in children. The pathophysiology of BBC is still undetermined. It is claimed that the inflammation is caused by the deep periodontal pocket caused by tilted tooth. Other authors claim that the reason behind the cyst formation could be because of a defect in the tooth eruption, which may lead to inflammation, proliferation of the epithelial cells and cyst formation ^1– 6^. It is also postulated that enamel projections at the area covered by reduced enamel epithelium between the cementoenamel junction and the furcation of the tooth could lead to cyst formation ^7^. It is worth mentioning that the etiopathogenesis of BBC is the same of paradental cyst, according to World Health Organization Classification of Head and Neck Tumours (2017).

Clinically, bilateral BBC has few challenging similarities with dentigerous cyst, and paradental cyst. For example, all previously mentioned cysts are commonly associated with a tooth. However, paradental cysts and dentigerous cysts usually occur in adults in the mandibular third molar region ^8, 9^. Dentigerous cyst is classified as a developmental cyst that may also occur in the mandibular first molar areas and sometimes interfere with the process of normal tooth eruption in children. On the other hand, paradental cyst is considered as an inflammatory cyst, which commonly occurs in the lateral surface of a vital tooth usually the first and second mandibular molars of primary teeth.

Radiographic interpretation is essential to assist recognize and differentiate between different cystic lesions. Normally cystic lesions have a well-defined, unilocular radiolucency with a sclerotic margin; however, cysts sometimes present with different features. For instance, paradental cysts present on the lateral crown of an incompletely erupted tooth, while dentigerous cysts usually surround the crown of an unerupted tooth. Every rule has an exception, it is challenging to differentiate between the circumferential dentigerous cyst and the BBC radiographically as both illustrated as radiolucent cysts involving a completely unerupted tooth. Histopathologically, the cyst wall under the microscope shows chronic inflammatory lymphocytes and two to four cell layers thick of proliferating lining of nonkeratinized stratified squamous epithelium.

The diagnosis of BBC should be established based on the clinical, radiographical and microscopic characteristics. The clinical examination, radiographic interpretation and histopathologic analysis of both lesions displaying cystic origin with bilateral buccal expansion involving both mandibular first molars in a seven-year-old child associated with bilateral BBC was presented here. This reported case had similar clinical, radiographical and histopathological features of bilateral mandibular BBC. It is worth mentioning that the case presented here is very rare because it describes bilateral mandibular BBC. To the best of our knowledge, the bilateral characteristic of BBC is very rare; there are only few cases have been reported in the English-language literature. In this article, we reviewed 16 manuscripts of bilateral mandibular BBC, reporting a total of 20 cases in the period between 1970 to 2019. Furthermore, these cases occurred in different ages starting from 4 years to 13 years with predilection to male patients ( Table 1).

**Table 1.** Data of reported cases with Bilateral mandibular buccal bifurcation cysts since 1970.

Author	Year	Patient age	Patient gender	Number of bilateral cases	Associated tooth	Degree of tooth eruption	Management	Follow up
Stanback ^10^	1970	9 years	M	1	Bilateral mandibular permanent first molars/vital	Not erupted	Marsupialization	2 years
Swerdloff ^11^	1980	7 years	Not reported	1	Not reported	Not reported	Enucleation	6 months
Vedtolfe and Praetorius ^12^	1989	13 years	F	2	Case1: Bilateral mandibular permanent second molars/vital	Fully erupted	Enucleation	1–6 years
		Not reported	Not reported		Case2: Bilateral mandibular permanent first molars/vital	Not reported	Enucleation	1–6 years
Packota *et al.* ^13^	1990	8 years	Not reported	1	Bilateral mandibular permanent first molars/vital	Partially erupted	Enucleation	6 months
Bohay *et al.* ^14^	1992	Not reported	Not reported	1	Bilateral mandibular permanent first molars/vital	Not reported	Enucleation	8 months
Martinez-Conde *et al.* ^15^	1995	11 years	M	1	Bilateral mandibular permanent second molars/vital	Partially erupted	Enucleation/tooth extraction	0
David *et al.* ^6^	1998	8 years	M	3	Case1: Bilateral mandibular permanent first molars/vital	Fully erupted	Regress without procedure	1–2 years
		9 years	M		Case2: Bilateral mandibular permanent first molars/vital	Not erupted	9 months follow up then enucleation right side/ no procedure left side	1–2 years
		7 years	M		Case3: Bilateral mandibular permanent first molars/vital	Fully erupted	Daily irrigation with saline and hydrogen peroxide	1–2 years
Shohat I *et al.* ^16^	2003	13 years	M	2	Case1: Bilateral mandibular permanent second molars/vital	Fully erupted	Enucleation/tooth extraction	2 years
		8 years	M		Case2: Bilateral mandibular permanent first molars/vital	Fully erupted	Enucleation/tooth extraction	2 years
Gallego ^17^	2007	8 years	M	1	Bilateral mandibular permanent first molars/vital	Fully erupted	Enucleation left side/no procedure right side	1 year
Corona-Rodriguez *et al.* ^18^	2011	7 years	M	1	Bilateral mandibular permanent first molars/vital	Erupted	No procedure right side/enucleation left side	6 months
Ramos *et al.* ^19^	2012	9 years	M	1	Bilateral mandibular permanent first molars/vital	Erupted	Enucleation	1 year
Borgonovo *et al.* ^20^	2012	8 years	M	1	Bilateral mandibular permanent first molars/vital	Not erupted	Enucleation	1 year
Boffano P *et al.* ^21^	2012	9 years	M	1	Bilateral mandibular permanent first molars/vital	Not reported	Enucleation	6 months
Issler A *et al.* ^22^	2013	8 years	F	1	Bilateral mandibular permanent first molars/vital	Not erupted	Enucleation	18 months
Bautista *et al.* ^23^	2019	7 years	F	1	Bilateral mandibular permanent first molars/vital	Left first molar was not erupted	Enucleation/bone graft	0
						Right first molar was partially erupted		
Present case	2020	7 years	M	1	Bilateral mandibular permanent first molars/vital	Not erupted	Enucleation/tooth extraction	1 year

M: Male; F: female

Through the past years different approaches have been performed to manage the BBC. In some cases, the way of management was only by following up the lesion with no interventions; some lesions showed different degrees of regression and others needed intervention ^6– 9, 18^. On the other hand, other cases underwent surgical enucleation of the cysts either with the involved tooth or maintaining the involved tooth. In the current case, we chose to perform a surgical procedure under general anesthesia to enucleate both bilateral mandibular BBCs with the involved teeth to reduce the risk of recurrence and to fulfill the patient’s parent’s desire.

## Conclusion

BBC typically affects children in the first decade of life. BBC occurs in the buccal area of the mandibular first molar. Only a few bilateral mandibular BBC cases were reported in the literature. Although bilateral mandibular BBC is uncommon, the diagnosis would be less challenging if it is established by the correlation of the clinical examination, radiographic interpretation and histopathological analysis. Moreover, the clinical features of bilateral mandibular BBC summarized in this review could assist specialists to an accurate diagnosis and provide patients with optimal management.

## Patient perspective

The parents mentioned that the surgery has an excellent impact on their son’s life. Also, the outcomes met their expectations as the facial swellings disappeared.

## Consent

Written informed consent was obtained from the patient’s father for publication of this case report and accompanying images.

## Data availability

All data underlying the results are available as part of the article and no additional source data are required.
