# Supplementary material for: Case Report: Bilateral mandibular buccal bifurcation cysts
Source: F1000Res. 2021 Feb 19;9:1502. Originally published 2020 Dec 23. [Version 3] doi: 10.12688/f1000research.28000.3 (PMC7879215; doi:10.12688/f1000research.28000.3)
Supplement: Peer review history

# Peer review: Reviewer response for version 3

**DOI:** 10.5256/f1000research.54764.r79944

**Published:** 2021-02-24

**Authors:** Konstantinos I. Tosios

**Affiliations:** 1 Department of Oral Medicine and Pathology, School of Dentistry, National and Kapodistrian University of Athens, Athens, Greece

My previous comments were not answered by the authors. Therefore, I suggest for the manuscript "Approved with major reservations".

Are enough details provided of any physical examination and diagnostic tests, treatment given and outcomes?

Partly

Is the case presented with sufficient detail to be useful for other practitioners?

No

Is sufficient discussion included of the importance of the findings and their relevance to future understanding of disease processes, diagnosis or treatment?

No

Is the background of the case’s history and progression described in sufficient detail?

Partly

Reviewer Expertise:

Oral Pathology

I confirm that I have read this submission and believe that I have an appropriate level of expertise to confirm that it is of an acceptable scientific standard, however I have significant reservations, as outlined above.

# Peer review: Reviewer response for version 2

**DOI:** 10.5256/f1000research.54072.r79130

**Published:** 2021-02-11

**Authors:** Shadia Abdel-Hameed Elsayed

**Affiliations:** 1 Faculty of Dental Medicine for Girls, Al Azhar University, Cairo, Egypt; 2 College of Dentistry, Taibah University, Medina, Saudi Arabia

Thank you for this interesting, well written case study.

- The table of cases from past literature is very informative.
- Some points need to be explained.
- Please add the prevalence rate of such unusual cases to the introduction with citation.
- To evaluate the lesion extent, was CT or CBCT performed prior to surgery?  If so, please include a photograph of representing cut.
- Please also include an extraoral clinical photograph.
- Add details on the prescribed drugs and instructions for postoperative care.
- Please address previous citations regarding surgical management of this unusual cyst with complete tooth removal in the discussion.

Are enough details provided of any physical examination and diagnostic tests, treatment given and outcomes?

Partly

Is the case presented with sufficient detail to be useful for other practitioners?

Yes

Is sufficient discussion included of the importance of the findings and their relevance to future understanding of disease processes, diagnosis or treatment?

Partly

Is the background of the case’s history and progression described in sufficient detail?

Yes

Reviewer Expertise:

Oral & Maxillofacial Surgery

I confirm that I have read this submission and believe that I have an appropriate level of expertise to confirm that it is of an acceptable scientific standard.

# Response

**Published:** 2021-02-15

**Authors:** Ashwag Aloyouny

**Affiliations:** Princess Nourah Bint Abdulrahman University, Saudi Arabia

- Please add the prevalence rate of such unusual cases to the introduction with citation

             (Done)

- To evaluate the lesion extent, was CT or CBCT performed prior to surgery?  If so, please include a photograph of representing cut.

             No CT or CBCT were performed.  

- Please also include an extraoral clinical photograph.

            No extraoral photograph was taken 

- Add details on the prescribed drugs and instructions for postoperative care.
  (Done)

# Peer review: Reviewer response for version 2

**DOI:** 10.5256/f1000research.54072.r77129

**Published:** 2021-02-02

**Authors:** Konstantinos I. Tosios

**Affiliations:** 1 Department of Oral Medicine and Pathology, School of Dentistry, National and Kapodistrian University of Athens, Athens, Greece

This is a case of bilateral buccal bifurcation cysts that is unusual in that (a) it caused an extensive esthetic disfiguration leading to “social exclusion” and (b) necessitated extraction of two unerupted permanent molar teeth. However, as there are no clinical photographs, intra-oral or extra-oral, the first feature cannot be documented. Even more, the authors do not contemplate on the reasons that dictated extractions: does the risk of recurrence (how common is it?) balance the loss of two permanent first molars? A more thorough discussion is necessary.

 More specific comments:

**Introduction**

- The Introduction contains information (i.e. “Children between the age of 4 and 14 years are most commonly affected.”) not supported be relevant references.

**Clinical Presentation**

- The swelling are described as “painful” by the patient’s guardian. It would be interesting to know whether those swellings were, also, painful on palpation and/or showed pus excretion, or whether the authors noticed any orifice on the mucosa through which they could probe the cyst.
- The teeth are described as “ unerupted incomplete permanent mandibular first molars” (Case presentation, paragraph 3). Do they mean partially/incompletely erupted, as they were clinically not evident but radiographically not surrounded by bone?
- The histopathologic description should be reorganized: “multiple pieces of cyst wall” are profoundly consistent with “cystic lesions”, while it is the presence “of non-keratinized stratified squamous epithelium”, not “bland looking spindle-shaped fibroblasts and degenerated bony spicules” that make the findings “consistent with cystic lesions”. Fig. 3 is not of proper quality.

**Discussion**

- The Discussion contains information not supported be relevant references.
- The main aim of Discussion is for the authors to discuss their findings and offer (if possible) new insights. For instance, the authors correctly state that the inflammation caused by a deep periodontal pocket, or a defect in tooth eruption are possible reasons for the development of . Which of those factors possibly caused the lesions in their cases? How do they explain the bilaterality of the lesion?

- The authors should be very careful in what they state. For instance, in the 2 ^nd^ paragraph they write:
  - “Clinically, bilateral BBC has few challenging similarities with dentigerous cyst, and paradental cyst.” Only bilateral BCC has those similarities, not the usual unilateral variant?  
  - “all previously mentioned cysts are commonly associated with a tooth”. This is common for most cysts, inflammatory or developmental!
  - “paradental cysts and dentigerous cysts usually occur in adults in the mandibular third molar region” and later “paradental cyst… which commonly occurs in the lateral surface of a vital tooth usually the first and second mandibular molars of primary teeth.” Paradental cyst usually develops distally to the third permanent molar.
- “Radiographic interpretation is essential to assist recognize and differentiate between different cystic lesions”. Please correct syntaxis.
- “on the lateral crown”. Wrong expression.
- “Every rule has an exception, it is challenging to differentiate between the circumferential dentigerous cyst and the BBC radiographically as both illustrated as radiolucent cysts involving a completely unerupted tooth”. The authors correctly include this variety of dentigerous cyst in the d.d. of the present case (why don’t they state it in the case presentation), but they should give more information not only for the shake of completeness of their work, but also for “tutorial” reasons. The “Every rule has an exception” does not to fit in that case, while the whole phrase needs reconsideration.
- “Histopathologically, the cyst wall under the microscope shows chronic inflammatory lymphocytes and two to four cell layers thick of proliferating lining of nonkeratinized stratified squamous epithelium”. Repetition of well known information. It would more interesting to know if histopathologic examination can differentiate the cysts included in the d.d.
- “The diagnosis of BBC should be established based on the clinical, radiographical and microscopic characteristics. The clinical examination, radiographic interpretation and histopathologic analysis of both lesions displaying cystic origin with bilateral buccal expansion involving both mandibular first molars in a seven-year-old child associated with bilateral BBC was presented here. This reported case had similar clinical, radiographical and histopathological features of bilateral mandibular BBC. It is worth mentioning that the case presented here is very rare because it describes bilateral mandibular BBC. To the best of our knowledge, the bilateral characteristic of BBC is very rare; there are only few cases have been reported in the English-language literature.” This paragraph says the same thing many times. Please reconsider.

Are enough details provided of any physical examination and diagnostic tests, treatment given and outcomes?

Partly

Is the case presented with sufficient detail to be useful for other practitioners?

No

Is sufficient discussion included of the importance of the findings and their relevance to future understanding of disease processes, diagnosis or treatment?

No

Is the background of the case’s history and progression described in sufficient detail?

Partly

Reviewer Expertise:

Oral Pathology

I confirm that I have read this submission and believe that I have an appropriate level of expertise to confirm that it is of an acceptable scientific standard, however I have significant reservations, as outlined above.

# Peer review: Reviewer response for version 2

**DOI:** 10.5256/f1000research.54072.r77849

**Published:** 2021-01-22

**Authors:** Denise Tostes Oliveira

**Affiliations:** 1 Department of Surgery, Stomatology, Pathology and Radiology, Area of Pathology, Bauru School of Dentistry, University of São Paulo, Bauru, Brazil

There are no suggestions. The manuscript is ok.

Are enough details provided of any physical examination and diagnostic tests, treatment given and outcomes?

Yes

Is the case presented with sufficient detail to be useful for other practitioners?

Yes

Is sufficient discussion included of the importance of the findings and their relevance to future understanding of disease processes, diagnosis or treatment?

Yes

Is the background of the case’s history and progression described in sufficient detail?

Yes

Reviewer Expertise:

Oral pathology

I confirm that I have read this submission and believe that I have an appropriate level of expertise to confirm that it is of an acceptable scientific standard.

# Peer review: Reviewer response for version 1

**DOI:** 10.5256/f1000research.30969.r77339

**Published:** 2021-01-18

**Authors:** Denise Tostes Oliveira

**Affiliations:** 1 Department of Surgery, Stomatology, Pathology and Radiology, Area of Pathology, Bauru School of Dentistry, University of São Paulo, Bauru, Brazil

This is an interesting and well-documented case report of bilateral buccal bifurcation cysts (BBC). It is important to include in the manuscript that the etiopathogenesis of BBC is the same of paradental cyst, according to World Health Organization Classification of Head and Neck Tumours (2017). In addition, the diagnosis should be established based in the clinical/radiographic and microscopic characteristics.

Are enough details provided of any physical examination and diagnostic tests, treatment given and outcomes?

Yes

Is the case presented with sufficient detail to be useful for other practitioners?

Yes

Is sufficient discussion included of the importance of the findings and their relevance to future understanding of disease processes, diagnosis or treatment?

Yes

Is the background of the case’s history and progression described in sufficient detail?

Yes

Reviewer Expertise:

Oral pathology

I confirm that I have read this submission and believe that I have an appropriate level of expertise to confirm that it is of an acceptable scientific standard, however I have significant reservations, as outlined above.
